# Characterization of Transcriptional Changes in ERG Rearrangement-Positive Prostate Cancer Identifies the Regulation of Metabolic Sensors Such as Neuropeptide Y

**DOI:** 10.1371/journal.pone.0055207

**Published:** 2013-02-04

**Authors:** Petra Massoner, Karl G. Kugler, Karin Unterberger, Ruprecht Kuner, Laurin A. J. Mueller, Maria Fälth, Georg Schäfer, Christof Seifarth, Simone Ecker, Irmgard Verdorfer, Armin Graber, Holger Sültmann, Helmut Klocker

**Affiliations:** 1 Division of Experimental Urology, Department of Urology, Innsbruck Medical University, Innsbruck, Austria; 2 Oncotyrol, Center for Personalized Cancer Medicine GmbH, Innsbruck, Austria; 3 Institute for Bioinformatics and Translational Research, University for Health Sciences, Medical Informatics and Technology (UMIT), Hall in Tirol, Austria; 4 Unit Cancer Genome Research, Division of Molecular Genetics, German Cancer Research Center and National Center of Tumor Diseases, Heidelberg, Germany; 5 Division of Human Genetics, Department of Medical Genetics, Molecular and Clinical Pharmacology, Innsbruck Medical University, Innsbruck, Austria; University of Colorado, United States of America

## Abstract

ERG gene rearrangements are found in about one half of all prostate cancers. Functional analyses do not fully explain the selective pressure causing ERG rearrangement during the development of prostate cancer. To identify transcriptional changes in prostate cancer, including tumors with ERG gene rearrangements, we performed a meta-analysis on published gene expression data followed by validations on mRNA and protein levels as well as first functional investigations. Eight expression studies (n = 561) on human prostate tissues were included in the meta-analysis. Transcriptional changes between prostate cancer and non-cancerous prostate, as well as ERG rearrangement-positive (ERG+) and ERG rearrangement-negative (ERG−) prostate cancer, were analyzed. Detailed results can be accessed through an online database. We validated our meta-analysis using data from our own independent microarray study (n = 57). 84% and 49% (fold-change>2 and >1.5, respectively) of all transcriptional changes between ERG+ and ERG− prostate cancer determined by meta-analysis were verified in the validation study. Selected targets were confirmed by immunohistochemistry: NPY and PLA2G7 (up-regulated in ERG+ cancers), and AZGP1 and TFF3 (down-regulated in ERG+ cancers). First functional investigations for one of the most prominent ERG rearrangement-associated genes - neuropeptide Y (NPY) - revealed increased glucose uptake *in vitro* indicating the potential role of NPY in regulating cellular metabolism. In summary, we found robust population-independent transcriptional changes in prostate cancer and first signs of ERG rearrangements inducing metabolic changes in cancer cells by activating major metabolic signaling molecules like NPY. Our study indicates that metabolic changes possibly contribute to the selective pressure favoring ERG rearrangements in prostate cancer.

## Introduction

About one half of all prostate cancers harbor a gene rearrangement [1]. The latter is formed by fusion of 5′ regulatory elements of an androgen-regulated gene to the coding region of a member of the E twenty-six (ETS) gene family of transcription factors. *ETS* rearrangements result in androgen-driven over-expression of ETS transcription factors [1]. The most common ETS rearrangement is the translocation of the androgen-regulated transmembrane protease serine 2 (*TMPRSS2*) gene, with the v-ets erythroblastosis virus E26 oncogene homolog gene (*ERG*) transcription factor accounting for about 85% of all ETS rearrangement-positive prostate cancers [2]–[4]. ERG rearrangement is an early event in the genesis of prostate cancer. It is already present in local low-grade prostate cancer [5], [6] and persists in metastatic and castration-resistant types (CRPC) [1], [4], [7]. The early appearance and the high frequency of ERG rearrangements indicate the selective benefit of rearrangement-positive cells in prostate cancer.

Functional analyses performed thus far have not provided a comprehensive explanation for the selective pressure forcing ERG rearrangement in early stages of prostate cancer. ERG rearrangement results in ERG overexpression [1]. The latter was reported to promote cancer cell migration and invasion as well as cellular dedifferentiation and transformation [8]–[11]. The role of ETS rearrangement in the progression of prostate cancer has also not been clarified. While some studies indicate an association between rearrangement-positive cancers, more aggressive tumors and a poor prognosis (i.e. [12]–[14]), others report no such association (i.e. [15]–[17]); some even report a favorable prognosis (i.e. [18], [19]). We obviously need more information about ETS rearrangement-positive prostate cancers in order to understand the biology of prostate cancer.

A large body of gene expression data has been published since the procedure of expression analysis using microarrays was established more than a decade ago [20]. These studies have reported a variety of alterations in gene expression associated with various diseases, including prostate cancer. Validation of the large quantity of data obtained from gene expression experiments is challenging. Just a small number of the identified candidates have been functionally validated. These data are far from exhaustive and still contain a lot of information awaiting exploitation. Meta-analyses permit combined analyses of individual studies, are less influenced by local findings, and allow reduction of data to achieve robust results.

We present a meta-analysis on published gene expression data with a view to identifying transcriptional changes in prostate cancer. Our approach included comparison of prostate cancer versus benign prostate tissue, and ERG rearrangement-positive (ERG+) to ERG rearrangement-negative (ERG−) prostate cancers. We validated the results of our meta-analysis using data from an independent microarray study and confirmed selected targets by immunohistochemistry. We also performed preliminary functional investigations for one of the most prominent regulated genes, namely neuropeptide Y (NPY). Our results indicate that ERG-rearrangements possibly induce metabolic changes in cancer cells by activating major metabolic signaling molecules such as NPY.

## Materials and Methods

### Sample Cohorts

Tissue samples used for the meta-analysis are described elsewhere (studies and references in Table 1). Tissue samples for the validation expression study and immunohistochemical studies were selected from the Innsbruck prostate cancer biobank. This biobank was established in the course of the Tyrolean early detection program for prostate cancer at the Department of Urology, Innsbruck Medical University [21]. Written consent was obtained from all patients and documented in the database of the University Hospital Innsbruck in agreement with statutory provisions and the requirements of the ethics committee of the Innsbruck Medical University. The study was approved by the ethics committee of the Innsbruck Medical University (Study no. AM 3174, amendment 2). Cohorts analyzed here were the following: a) Expression analysis validation study; 57 prostate cancer tissues; GSC 5 n = 1, GSC 6 n = 5, GSC 7 n = 36, GSC 8 n = 3, GSC 9 n = 11, GSC 10 n = 1; patients’ age, mean ± standard deviation (SD), 61.7±6.9 years; patients’ serum prostate specific antigen (PSA) level, 7.4±5.0 ng/ml. b) Immunohistochemical study; 93 prostate cancer tissues, GSC 5 n = 19, GSC 6 n = 22, GSC 7 n = 32, GSC 8 n = 10, GSC 9 n = 10; patients’ age, mean ± standard deviation (SD), 60.6±6.4 years; PSA level, 6.6±5.4 ng/ml.

**Table 1:** Gene expression studies included in the meta-analysis.

	Study	Cancer (CA) vs. benign (BE)		ERG+ vs. ERG−		Ref.	Source*	Platform
		n** CA	n BE	n ERG+	n ERG−			
1	Bermudo	21	8			[68]	AE E-MEXP-1331	Affymetrix GeneChip Human Genome Focus Array
2	Chandran	57	15	20	19	[69]	GEO GSE-6919	Affymetrix GeneChip Human Genome U95Av2
3	Liu	41	13	14	13	[70]	AE E-TABM-26	Affymetrix GeneChip Human Genome HG-U133A
4	Singh	50	48	17	16	[71]	O Singh Prostate	Affymetrix GeneChip Human Genome U95Av2
5	Tsavachidou	23	49	8	7	[72]	AE E-MEXP-1327	Affymetrix GeneChip Human Genome HG-U133A
6	Varambally	7	6			[73]	GEO GSE3325	Affymetrix GeneChip Human Genome U133 Plus 2.0
7	Wallace	68	14	23	22	[74]	GEO GSE6956	Affymetrix GeneChip Human Genome U133A 2.0
8	Wang	138	3	47	46	[75]	GEO GSE8218	Affymetrix GeneChip Human Genome HG-U133A
	**Total**	**405**	**156**	**129**	**123**			

The Affymetrix microarray technology was used in all of the studies.

* AE, Array Express; GEO, Gene Expression Omnibus; O, Oncomine; status 10/2010.

** n, number of samples used for the meta-analysis; samples that did not fulfill the quality criteria were excluded.

### Meta-analysis

We selected eight expression studies, comprising 561 human prostate tissues, for the meta-analysis (Table 1, Figure S1). These are listed in the databases Gene Expression Omnibus (http://www.ncbi.nlm.nih.gov/geo) [22], Array Express (http://www.ebi.ac.uk/arrayexpress) [23], and Oncomine (http://www.oncomine.org) [24]. All studies were performed using the Affymetrix microarray technology.

For integrative analysis of microarray data, raw data, as stored in CEL files, was normalized using gcRMA algorithm [25], [26]. For detailed information about study-specific data preprocessing, see supplementary methods. To perform a cross-study comparison of gene expression levels, platform-specific gene probe-set identifiers were mapped to a common namespace, as previously described [27], [28]. Here the platform-specific identifiers were mapped to Entrez gene identifiers using the current probeset/Entrez mappings from BioMart via the biomaRt package [29]. Wherever more than one probe-set was mapped to an Entrez gene identifier, the probe-set with the highest variance was used for analysis. To identify differentially regulated genes we used a two-step approach. First, we derived combined p-values across the studies using Fisher’s inverse chi-squared method [30]. We then calculated combined (weighted) fold changes. In a previous study, the authors used a permutation test to assess significance [27]. We, on the other hand, derived the information from a chi-squared distribution as suggested in [31]. See supplementary methods for details about p-values and fold-change calculations. Functional annotation clustering of the results of meta-analysis was performed using the DAVID database (http://david.abcc.ncifcrf.gov) [32].

### Validation Expression Analysis

An independent microarray data set (GSE32571) previously generated by coauthors was used for validation of the ERG-associated gene signature. For the present analyses, prostate cancer tissues (n = 57) were assigned to the groups ERG+ and ERG− using a break-apart fluorescent in situ hybridization (FISH) assay, as described earlier [33]. Tissue samples were isolated by macrodissection from 10-µm cryosections. Total RNA was isolated with the EZ1 RNA Mini Kit (Qiagen) according to the manufacturer’s instructions, checked for quality using the Agilent 2100 Bioanalyzer (Agilent Technologies), and quantified. Total RNA was prepared for hybridization on Illumina Human Sentrix-12 BeadChip arrays (Illumina) according to the manufacturer’s recommendations. Illumina microarray data were processed using the open source pipeline “Lumi” [34]. After quantile normalization, differential expression of genes was determined using the R package “LIMMA” [35]. Detailed methods and clinical data of the expression validation study are described elsewhere [36].

### Immunohistochemistry

Tissue slides (n = 10) and tissue microarrays (TMAs; n = 92; diameter 0.6 mm) containing cancer (n = 3) and benign (n = 1) cores of prostate tissue from prostate cancer patients were used for immunohistochemical analysis. All tissue samples were stained for ERG. Nine samples were excluded because of their small quantity of tumor tissue. 24 tissues showed very weak or heterogeneous ERG staining, whereas 69 tissues with intermediate to strong or negative ERG staining were assigned to the groups ERG+ and ERG−, respectively. 61 tissue samples were used for immunohistochemical comparison of ERG+ and ERG−. Antigen retrieval and immunohistochemistry were performed using a Discovery XT automated slide-staining system (Ventana Medical Systems). All target antibodies were tested on a test tissue microarray containing different human tissue samples. Immunohistochemical staining was evaluated by an experienced uropathologist (G.S.). Optimal antigen retrieval conditions were established for all target antibodies. Target antibodies, suppliers, article numbers, and concentrations used were as follows: anti-AZGP1, Sigma, HPA-012582, 1∶50; anti-ERG, Epitomics, EPR3864, 1∶100; anti-GPR116, Imgenex, IMG-71635, 1∶100; anti-neuropeptide Y, Abcam, ab30914, 1∶200; anti-PLA2g7, Sigma, HPA-0035915, 1∶400; anti-HPGD, Atlas, HPA004919, 1∶75; anti-TFF3, Strategic Diagnostics, 29940002, 1∶5000. Antigen retrieval for all antibodies was performed by heat pretreatment at 98°C for 1 hour in CC1, a tris-based buffer with a slightly basic pH. Target antibodies were incubated for 1 hour at 37°C, followed by iView DAB detection (diaminobenzidine visualization, Ventana Medical Systems) and hematoxylin counterstaining. Images were acquired using an Axio Imager M1 microscope (Zeiss) and TissueFAXS software (TissueGnostics). Quantitative immunohistochemical analysis was performed using the HistoQuest immunohistochemistry analysis software (TissueGnostics), which is a cell-based staining intensity analysis tool employing a nuclear cellular identification marker (in this case hematoxylin), followed by quantitative analysis of a given marker labeled in a different color (in this case cytoplasmatic staining with diaminobenzidine, DAB, brown).

### Cell Culture Experiments

DU145, DUCaP, LNCaP, PC3 and VCaP are derivatives of prostate cancer metastases. These were purchased from ATCC and cultured according to ATCC recommendations. EP156T and RWPE-1 are immortalized benign prostate epithelial cells, whereas CAF and PM151T are prostate stromal cells [37]–[39]. Benign and stromal cells were cultured as described earlier [37]–[39]. The identity of cancer cell lines was confirmed by forensic DNA fingerprinting methods employing the AmpFlSTR® SGM Plus® PCR amplification kit (Applied Biosystems).

Cells were seeded in 24-well plates, serum starved, and treated with 25 nM recombinant NPY/24 h (Sigma) for a total period of 48 hours. Total cell numbers were determined using the CASY cell counter and analyzer system (Schärfe System). Glucose levels were measured in cell culture supernatants using the Gluc Cell glucose monitoring system (Thermo Fisher Scientific). qPCR and immunoblot were performed as described earlier [40].

### Statistics

Statistical calculations for meta-analysis and validation expression analysis were conducted using the R (http://www.r-project.org), packages from Bioconductor open source bioinformatics database [41] and functions of MADAM [42].

Statistical calculations for immunohistochemistry and cell culture experiments were performed using SPSS 18 for Windows. The Kolmogorov–Smirnov test was used to investigate normal distribution of data sets. Non-normally distributed data and data with Gaussian (normal) distribution were analyzed using the Mann–Whitney U-test and Student’s T-test, respectively, in order to calculate the significance of differences between groups. P values below 0.05 were considered significant. Error bars in the histograms represent the standard deviation (SD) of a minimum of three independent experiments.

## Results

### Meta-analysis on Published Gene Expression Data for Prostate Cancer

**Figure 1:**
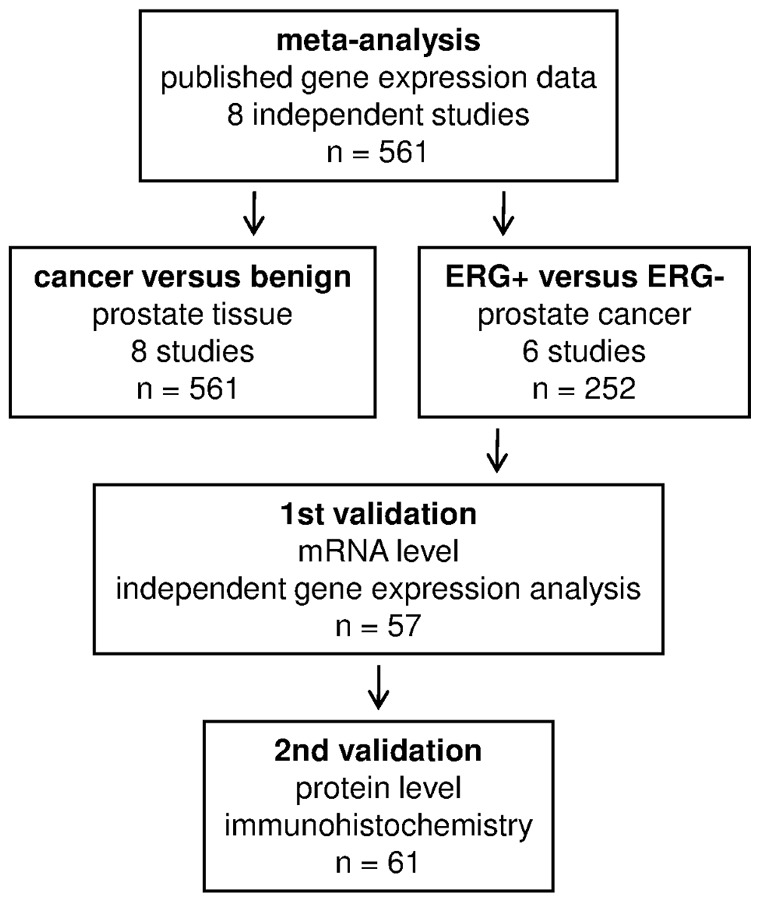
Study protocol. Meta-analysis was performed on eight independent gene expression studies focusing human prostate tissues. Two types of comparative analyses were used. Genes showing differentially regulated ERG+ and ERG− prostate cancer tissues were validated using an independent gene expression analysis (first validation) and immunohistochemical staining (second validation; only for selected genes).

To investigate changes in gene expression in human prostate cancer, we performed a meta-analysis of published gene expression data. Eight independent microarray studies were included in the meta-analysis, which consisted of 561 prostate tissue samples (Table 1). The following two issues were investigated: a) comparison of gene expression in prostate cancer tissues and benign prostate tissues; and b) comparison of gene expression in ERG rearrangement-positive (ERG+) and ERG rearrangement-negative (ERG−) prostate cancers (study plan in Figure 1).

**Figure 2:**
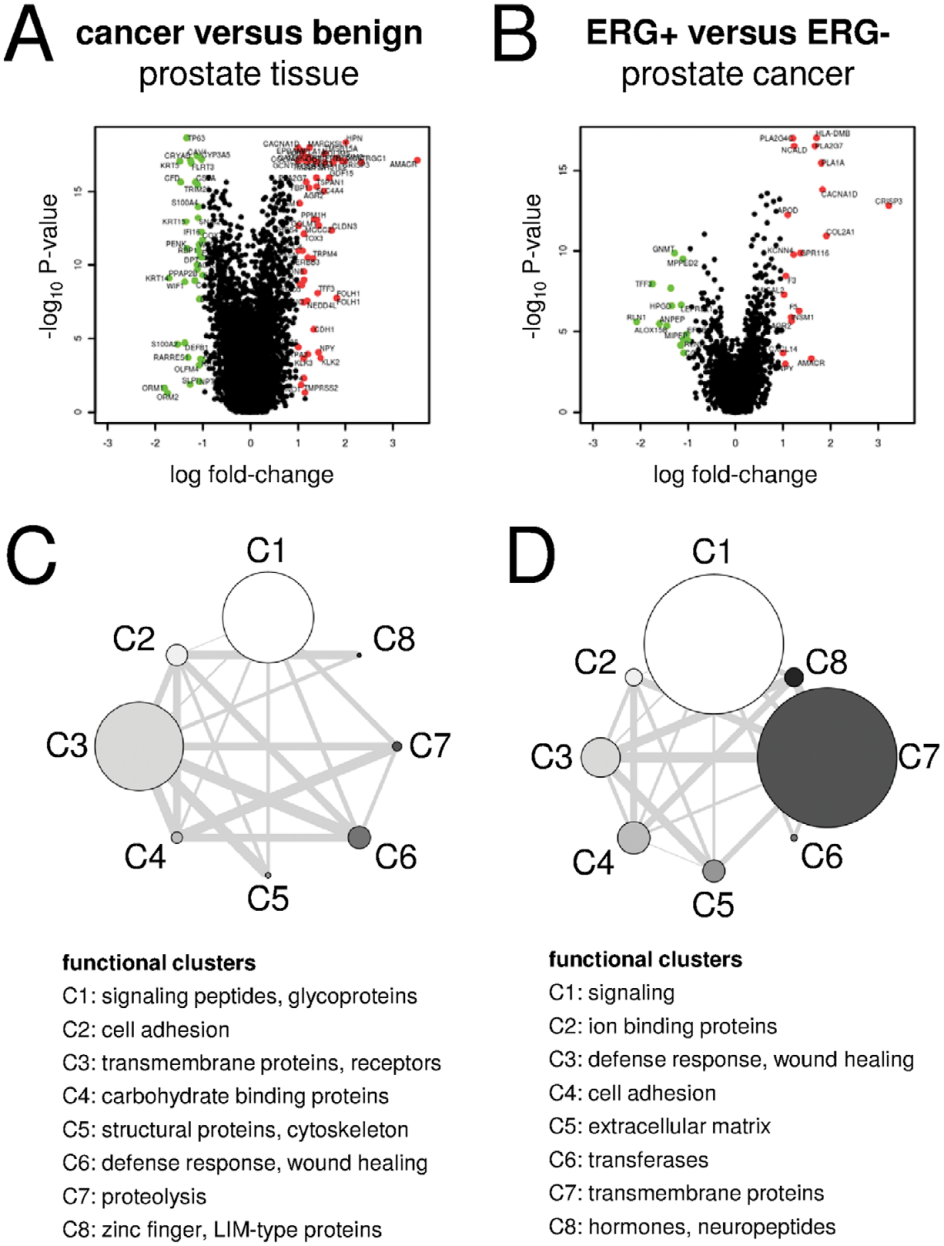
Results of the meta-analysis. Genes differentially regulated in prostate cancer and benign prostate tissue (A, C), and ERG+ and ERG− prostate cancer tissue (B, D). A–B) volcano plots. Differentially regulated genes were highlighted when at least 2-fold down- (green) or up-regulated (red), and had an adjusted p-value smaller than 0.1. C–D) Graphic diagram of the eight top-ranked functional clusters determined by functional annotation clustering, using the DAVID database. The size of the clusters correlates with the number of identified proteins associated with functional annotation (fold-change >1.5). When proteins are present in more than one cluster, the clusters are connected by lines. The thickness of the connecting lines reflects the number of proteins present in both connected clusters. Data concerning 561 tissue samples were used for the meta-analysis.

The results of our meta-analysis are shown in Figures 2A–B, and listed in the Table S1A–B. Results of the meta-analysis and the individual studies may also be viewed online at http://prostatedb.eigenlab.net/. In addition to the less conservative Chi-squared method, which we applied for deriving a summary p-value [31], the database also lists a more conservative summary p-value derived by a permutational approach [27].

We first compared prostate cancer tissues with benign prostate tissues. With regard to genes, which were at least 1.5-fold regulated and revealed an adjusted p-value <0.1, we found that 280 genes were up-regulated (46 of them more than 2-fold) while 275 genes were down-regulated (36 more than 2-fold) in prostate cancer tissues compared to benign prostate tissues (Figure 2A, Table S1A). Functional annotation revealed that differentially regulated genes (fold-change >1.5) code for signaling molecules (trans-membrane and extracellular signaling proteins), structural proteins (cytoskeleton and cell adhesion proteins) and proteins involved in proteolysis and wound healing (Figure 2C). Proteins known to be associated with prostate cancer (e.g. AMACR [43], AGR2 [44], CRISP3 [45], HPN [46], HOXC6 [47], OR51E2 [48]) as well as proteins not associated with prostate cancer thus far (examples PPM1H, SLC4A4, CAMKK2) were identified in the meta-analysis.

We then compared ERG+ and ERG− prostate cancer tissues. No information was available concerning the ERG rearrangement status of the prostate cancer samples used in the studies of the meta-analysis. ERG rearrangement results in ERG over-expression and is observed in about 50% of all prostate cancer tissues [1], [3]. We divided all prostate cancer samples of the studies included in the meta-analysis into three groups, based on their ERG expression level: ERG overexpression-positive samples, ERG intermediate samples, and ERG overexpression-negative samples (Figure S2). Each group consisted of one third of all samples of one study. ERG overexpression-positive samples were assumed to be ERG rearrangement-positive and assigned to the ERG+ category, while ERG overexpression-negative samples were assumed to be ERG rearrangement-negative and assigned the ERG− category. Samples assigned to the ERG intermediate group were excluded from the analysis to ensure accurate comparison of ERG+ and ERG− prostate cancer samples. The use of ERG expression as a surrogate for gene rearrangement-status has been described earlier [49] and was verified in our own microarray study as described below. The comparative meta-analysis revealed 109 up-regulated and 58 down-regulated genes (fold-change >1.5; adjusted p-value <0.1; 36 genes were more than 2-fold regulated) in ERG+ as compared to ERG− prostate cancer (Figure 2B, Table S1B). Differentially regulated genes were related to the functional clusters signaling (extracellular signaling peptides and hormone signaling), adhesion (cell adhesion and extracellular matrix proteins), and defense response (wound healing and inflammatory response; Figure 2D).

Our meta-analysis revealed changes in gene expression in various subgroups during the development of prostate cancer. A number of studies comprising independent patient cohorts were included in the analysis. Therefore, the identified alterations in gene expression signify general population-independent effects related to prostate cancer.

### Meta-analysis Validation by Independent Expression Analysis

**Figure 3:**
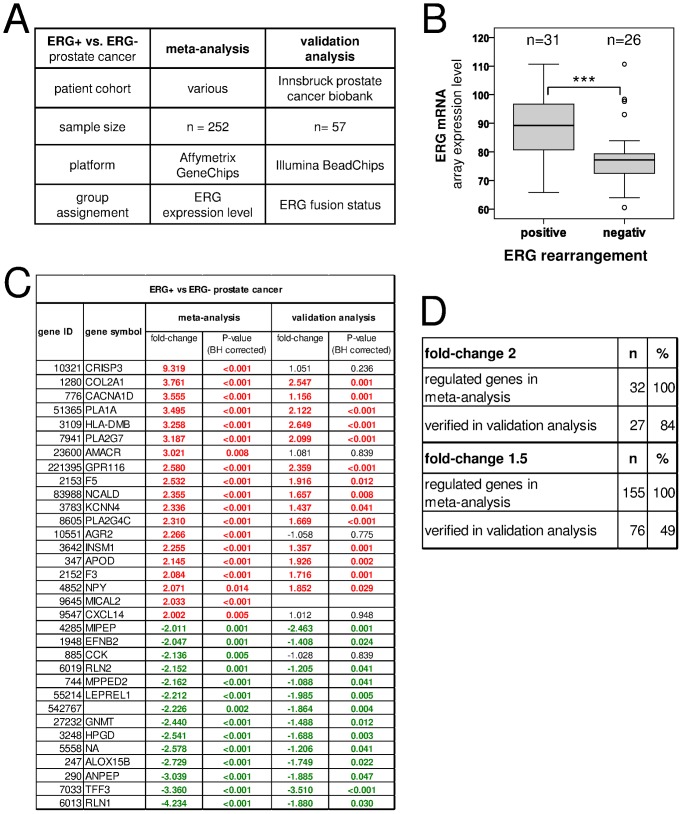
Validation of the meta-analysis. 84% of all genes found to be differentially regulated in ERG+ and ERG− tissues (fold-change >2) were verified by an independent expression study. A) Study characteristics. B) ERG expression levels in ERG rearrangement-positive and -negative tissue of the validation study. ERG rearrangements were determined by fluorescent in situ hybridization. C) Genes differentially regulated in ERG+ and ERG− tissues. D) Validated regulated genes in the meta-analysis and the validation study. P-value corrected BH; Fisher’s combined p-value, Benjamini-Hochberg corrected.

We then validated the results of our meta-analysis. We focused on a comparison of ERG+ and ERG− prostate cancers because these subtypes were described recently and have not been extensively investigated so far. For validation of the meta-analysis we used data from an independent expression study performed on an alternative expression platform (Illumina). The validation study differs from the meta-analysis studies in the following aspects: a) independent patient cohort (n = 57) selected from the Innsbruck prostate cancer biobank; b) alternative gene expression technology (validation study, Illumina BeadChip arrays; meta-analysis studies, Affymetrix GeneChip microarrays); and c) ERG+ and ERG− group assignment (Figure 3A). In the meta-analysis, ERG+ and ERG− tissues were assigned according to ERG expression levels. In the validation study the ERG rearrangement-status was determined by fluorescence in situ hybridization using a break-apart assay as described earlier [33] (Figure S3A).

We first confirmed that ERG rearrangement resulted in ERG over-expression (Figure 3B, Figure S3A–C), thus indicating that the ERG+ and ERG− groups were correctly assigned in the meta-analysis as well as the validation study. We then analyzed the expression of the 155 genes (12 genes were excluded because of missing or redundant probe sets) identified as being differentially regulated in ERG+ and ERG− tissues in the meta-analysis (FC >1.5; p<0.1). 49% (76/155) of all genes found to be differentially regulated in ERG+ and ERG− prostate cancer in the meta-analysis were verified in the validation study. When the validation analysis was confined to genes which were regulated at least 2-fold in the meta-analysis, these amounted to 84% (27/32) (Figure 3C–D, Table S2). When we analyzed the validation expression study as an independent study and investigated all genes in the arrays, these still amounted to 20% and 41% for FC>1.5 and FC>2, respectively (Table S2). The concordance between the meta-analysis and the independent validation study showed that our meta-analysis generated robust results which were independent of the sample cohort, sample assignment, and employed gene expression technology.

### Protein Analysis Using Immunohistochemistry

Our study had been focused on mRNA expression levels until this time. We then investigated whether proteins encoded by the differentially regulated genes are found in different quantities in ERG+ and ERG− prostate cancer tissues. We selected independent tissues (not used for expression analysis) from the Innsbruck prostate cancer biobank in order to further ensure that our investigation would reveal general ERG+ prostate cancer-related alterations rather than patient-specific differences.

To assign the tissues to the groups ERG+ and ERG−, we determined ERG protein levels by immunohistochemistry using an antibody previously specified for this application [50]. ERG immunohistochemistry permitted a clear distinction between ERG+ and ERG− tissues. In agreement with published data concerning ERG+ tissues, the staining intensity of ERG varied from strong to weak (Figure S4A) [50]. Some tissues appeared heterogeneous for ERG. Both ERG-positive and ERG-negative cancer cells were present in these tissues (Figure S4B). Staining controls were performed on a) benign prostate cells, which are negative for ERG (Figure S4C, left image); b) endothelial cells and lymphocytes, which stain positive for ERG [50] and constitute an internal staining control (example in Figure S4C, right image); and c) cell lines representing ERG+ (VCAP) or ERG− (Du145) prostate cancer (Figure S4D). In line with previous reports, approximately one half (here 60%) of all investigated prostate tissues appeared ERG-positive (summary of 93 prostate cancer cases in Figure S4E) [1], [3]. To reflect the meta-analysis, tissues with high to intermediate ERG staining were compared to tissues with negative ERG staining; tissues with heterogeneous and low ERG staining were excluded.

**Figure 4:**
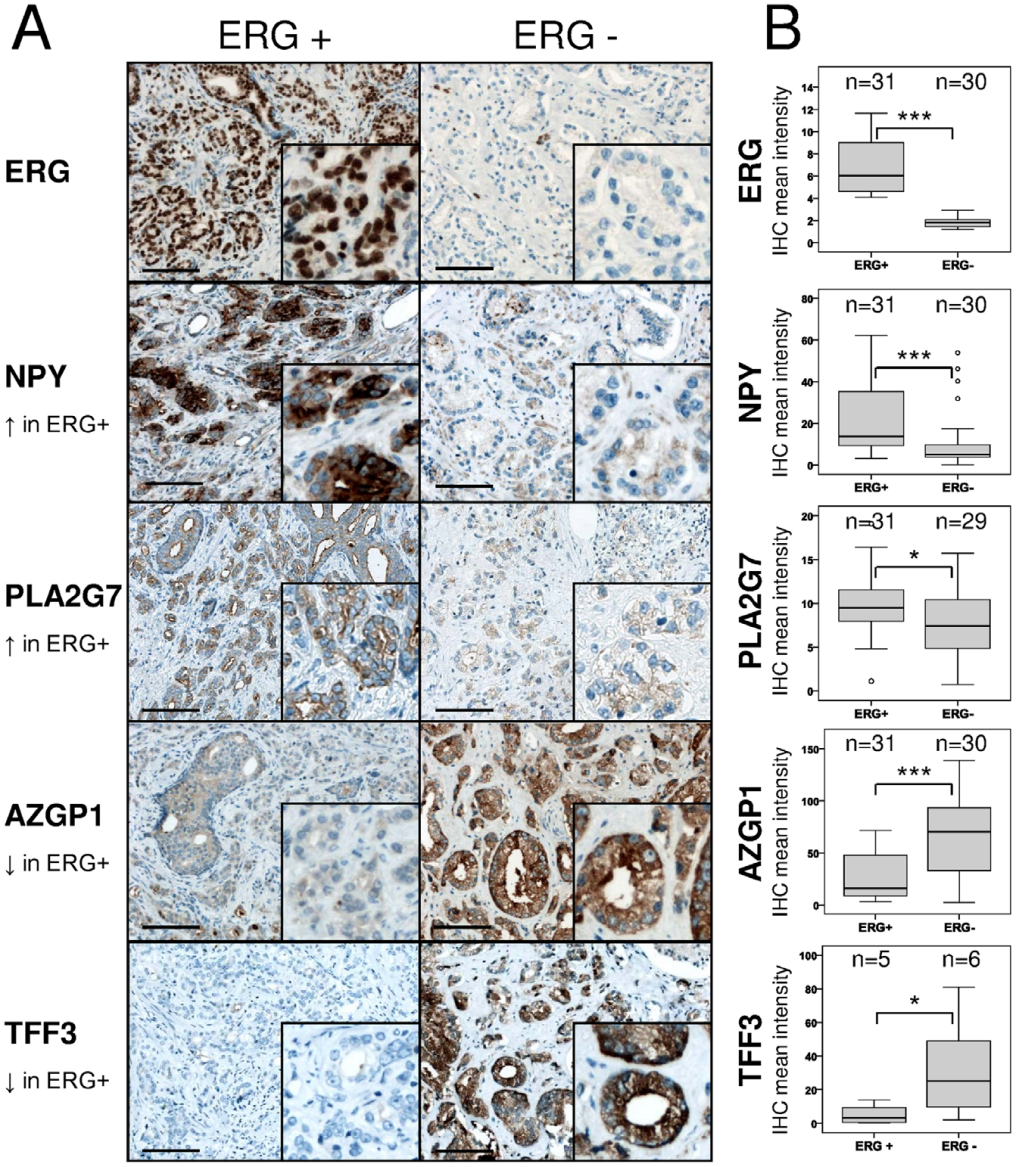
Immunohistochemistry for proteins coded by differentially regulated genes in ERG+ and ERG− prostate cancer. A) Consecutive slides of prostate tissues of two representative patients. B) Quantification of tissue specimens obtained from 61 different prostate cancer patients using the immunohistochemistry quantification software HistoQuest. HistoQuest does not distinguish between epithelial and stromal cells. Differences in staining intensity in epithelial cells exceed the differences shown in the box-blots. Bar, 100 µM. Statistics, Mann Whitney U-test; *,p<0.05; **p<0.01; ***p<0.001.

Six target genes were subjected to protein validation: *GPR116*, *NPY* and *PLA2G7*, three genes over-expressed in ERG+ tissues, and *AZGP1, HPGD* and *TFF3*, three genes down-regulated in ERG+ prostate cancer tissues. In four of the tested genes, the protein levels reflected the regulation of the mRNAs: NPY and PLA2G7 were up-regulated in ERG+ tissues while AZGP1 and TFF3 appeared down-regulated (Figure 4). TFF3 has been reported to be differentially regulated in ERG+ tissues [51]; therefore only 11 samples were used for comparison. In line with our data, PLA2G7 was recently reported to be related to ERG+ tumors [52]. Protein levels of the target genes *GPR116* and *HPGD* were not altered on comparison of ERG+ and ERG− prostate cancer (data not shown). It remains unclear whether the discrepancies observed between mRNA and protein levels of these two genes reflect biological (different regulation at mRNA and protein level) or technical (antibody performance) variations.

### ERG-associated Expression of NPY Induces Increased Glucose Uptake in Prostate Cancer Cells

To gain new insights about the role of ERG-rearrangements in prostate cancer, we investigated the functions of differentially regulated genes in ERG+ prostate cancer cells. NPY was selected for functional analysis. NPY is a small neuropeptide with was described as a central regulator of energy balance in the human body (reviewed in [53]). We measured glucose uptake *in vitro* to determine whether NPY induces metabolic changes in prostate cancer cells.

**Figure 5:**
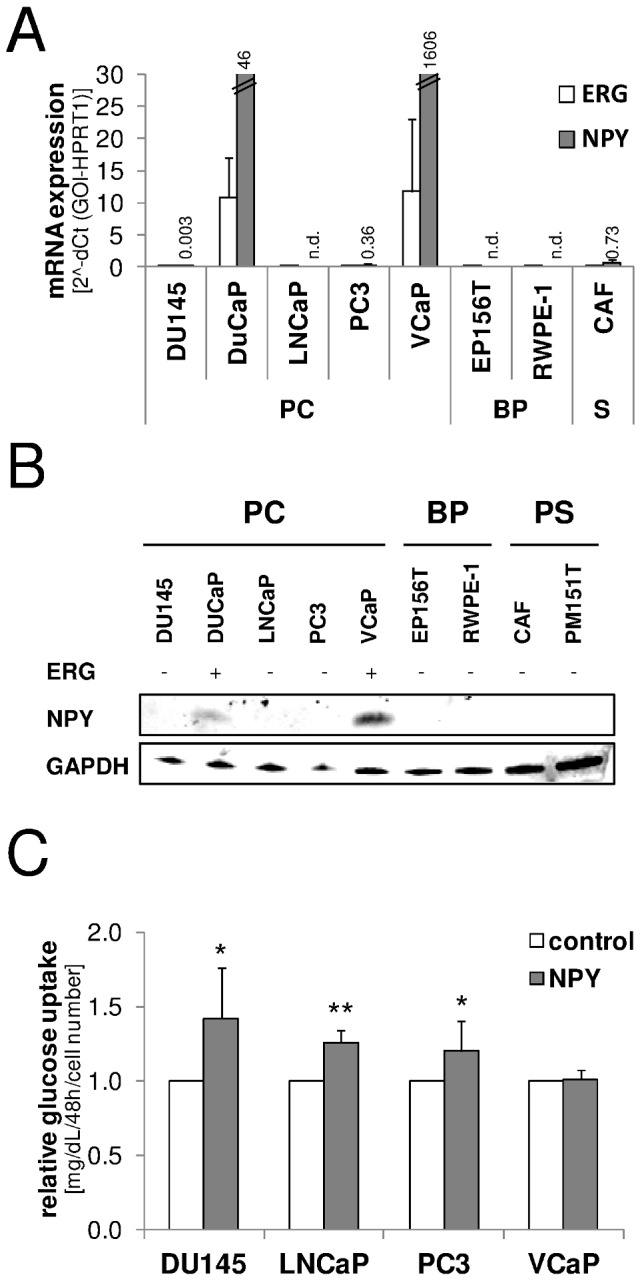
Neuropeptide Y (NPY) is up-regulated in ERG+ prostate cancer cell lines and increases glucose uptake. NPY was measured by qPCR (A) and immunoblot (B) in prostate cell lines. NPY expression was highest in ERG+ DuCAP and VCaP prostate cancer cells. C) NPY stimulation (48 h, 25 nM) increased glucose uptake in prostate cancer cells that do not express endogenous NPY (DU145, LNCaP, PC3). PC, prostate cancer cell lines; BP, benign prostate cell lines; PS, prostate stromal cell lines.

We confirmed NPY overexpression in ERG+ prostate cancer cell lines using qPCR and immunoblotting. qPCR expression levels of NPY revealed highest expression of NPY mRNA in the ERG+ cell lines DUCaP and VCaP compared to other prostate cell lines (Figure 5A). NPY protein was detectable by immunoblotting solely in DUCaP and VCaP (Figure 5B). When we treated prostate cancer cells which do not express endogenous NPY (DU145, LNCaP and PC3) with recombinant NPY (48 h, 25 nM), we observed greater glucose uptake in NPY-treated cells than in untreated cells (Figure 5C). This effect was not observed in cells expressing endogenous NPY (VCaP, Figure 5C). To investigate whether human prostate tissues are potentially NPY responsive, we finally compared the expression of NPY and the NPY-responsive receptors NPY1R, NPY5R and NPY2R (NPY affinity ranked, [54]) in the prostate with those in other human organs, using the Oncomine database (http://www.oncomine.org) [24]. We found NPY to be more abundant in benign and cancerous prostate tissue compared to benign and cancerous tissues derived from other organs while NPYRs were expressed to a similar extent in prostate and other tissues (representative studies are shown in Figure S5). Thus, several tissues might be NPY responsive. The prostate, however, produces significant levels of endogenous NPY. Taken together our data demonstrate that ERG-rearrangements in prostate cancer are associated with a variety of transcriptional changes in cancer cells including the expression of metabolic sensors like NPY.

## Discussion

This study was performed to determine changes in gene expression in prostate cancer, which are due to general alterations related to prostate and independent of patient cohorts, technical variations, and the applied methodology. We focused on transcriptional changes in ERG rearrangement-positive (ERG+) prostate cancer, because these tumors constituted a less-characterized subgroup of prostate cancers. We expected the results of this investigation to yield a publicly accessible database for gene expression alterations in prostate cancer and provide new insights into the biology of ERG+ prostate cancers. We observed, for the first time, that ERG rearrangements are possibly associated with metabolic changes in prostate cancer cells.

Our meta-analysis consisted of 561 tissues derived from eight independent studies. We performed two comparisons in the meta-analysis (cancer vs. benign prostate tissue and ERG+ vs. ERG− prostate cancer) so that the study would be of interest to a large scientific community. We validated the investigation using our own independent gene expression study performed on an independent patient cohort, employing an alternative gene expression method. The large numbers of validated genes (84%, comparison ERG+ vs. ERG−, cutoff of fold change >2) indicated that our meta-analysis had generated robust results. The alterations in gene expression in prostate cancer we describe here represent therefore general population-independent transcriptional changes in prostate cancer.

Interestingly, we identified many genes differentially regulated in ERG+ prostate cancer, which proteins were previously described as diagnostic or prognostic prostate cancer biomarkers, like AZGP1 [55], [56], APOD [57], [58], CRISP3 [45], NPY [59] and TFF3 [60]. Many of these putative prostate cancer markers were not successfully validated and translated into clinics. Our study indicates that these proteins possibly present ERG+ prostate cancer rather than general prostate cancer markers.

Transcriptional changes in prostate cancer probably exceed those identified in the present meta-analysis. The studies we used were published several years ago. The authors used gene chip technologies, which covered just a subset of all human genes. The stringent conditions required for a valuable meta-analysis led to further exclusion of several gene probes, which were measured in just a few studies or demonstrated high background variations. Finally, the tissue samples used in the studies were taken from whole tumor tissue, including stromal fractions. High stromal expression of certain genes may mask regulations occurring in epithelial tissue. Despite these limitations, published gene expression studies proved to be a very precious source of data, especially for meta-analysis. They were also useful for analyzing research questions the studies were not originally designed to address. Besides, much more information may yet be obtained from these studies.

We used the data to advance our current understanding of the biology of prostate cancer while focusing on the biology of ERG+ prostate cancer. Functional analyses performed thus far have been unable to fully explain the selective pressure forcing ERG rearrangement in early stages of prostate cancer. Apart from a pronounced migratory and dedifferentiated phenotype [8]–[11], ERG-related cancer-promoting functions remained largely undefined. We observed a variety of lipoproteins, phospholipases (e.g. APOD, PLA1A, PLA2G4C, PLA2G7), and several small secretory molecules with described metabolic functions (e.g. NPY, RLN1, RLN2) in our top-ranked ERG+ regulated genes. We speculated that ERG rearrangements induce metabolic changes in prostate cancer cells. We selected NPY, a factor highly overexpressed in ERG+ tumors, for functional analysis. NPY has been described as a potent orexigenic agent (reviewed in [53]). Investigations concerning the function of NPY in prostate cancer have been confined to proliferation and migration studies. These report weak effects of NPY on migration and cell-line-specific differences on cell proliferation [61]–[63]. When treating prostate cancer cells with NPY, we observed higher glucose uptake in NPY-treated cells. Our data indicate that NPY mediates metabolic functions in prostate cancer cells. As NPY is overexpressed in ERG+ prostate cancer cells, these data provide first signs of the fact that ERG rearrangements in prostate cancer possibly modulate metabolic functions through expression of metabolic regulators like NPY.

Apart of its function as an orexigenic agent, NPY was also shown to be involved in stress response, pain perception and regulation of bone homeostasis and turnover [64], [65]. As prostate cancer metastasizes preferentially to the bone, the latter is of special interest. Most prostate cancer bone metastases are characterized by a disrupted bone homeostasis and bone loss ( = osteolytic phenotype, for recent review see [66]). Disrupted bone homoeostasis and bone loss caused by reduced osteoblast activity were also described when elevated levels of NPY are present in the bone microenvironment (reviewed in [64]). NPY represses osteoblast activity. Thus, one major factor expressed by prostate cancer cells and modulating the bone microenvironment may be NPY. As NPY is highest expressed in ERG+ prostate cancers it would be interesting to investigate whether ERG+ differ from ERG− prostate cancer bone metastases. So far, data on ERG rearrangement and prostate cancer progression are very inconsistent, reporting a positive [12]–[14], [67], no [15]–[17] or even a negative [18], [19] correlation.

In the present study we investigated ERG rearrangement, which is the most common type of ETS rearrangement. Further studies will be needed to determine whether other ETS rearrangements involving ETV1, ETV4, ETV5 or ELK4 exert similar effects as ERG does.

In summary, the present study was focused on population-independent transcriptional changes in prostate cancer and give first hints that ERG rearrangement in prostate cancer induces metabolic changes. Selective pressures favoring ERG rearrangement in prostate cancer presumably include enhanced migration, invasion, and dedifferentiation of prostate cancer cells, as well as exert an impact on energy balance, availability and consumption.

## Supporting Information

Figure S1 **Prisma Flow Diagram.**
(PDF)

Figure S2 **Definition of ERG+ and ERG− prostate cancer tissues based on their ERG expression levels.**
(PDF)

Figure S3 **ERG rearrangement results in ERG overexpression.**
(PDF)

Figure S4 **ERG immunohistochemistry.**
(PDF)

Figure S5 **Expression of NPY and NPY-responsive receptors.**
(PDF)

Table S1 **Genes differentially regulated in prostate cancer determined in a meta-analysis of published gene expression data.**
(XLS)

Table S2 **Validation of genes differentially regulated in prostate cancer determined by meta-analysis using an independent expression analysis.**
(XLS)

Methods S1 **Meta-analysis: preparation of data, statistical calculations.**
(DOC)
