# Supplementary material for: Characterization of Transcriptional Changes in ERG Rearrangement-Positive Prostate Cancer Identifies the Regulation of Metabolic Sensors Such as Neuropeptide Y
Source: PLoS One. 2013 Feb 4;8(2):e55207. doi: 10.1371/journal.pone.0055207 (PMC3563644; doi:10.1371/journal.pone.0055207)
Supplement: Figure S1 — Prisma Flow Diagram. (PDF) [file pone.0055207.s001.pdf]

PRISMA 2009 Flow Diagram

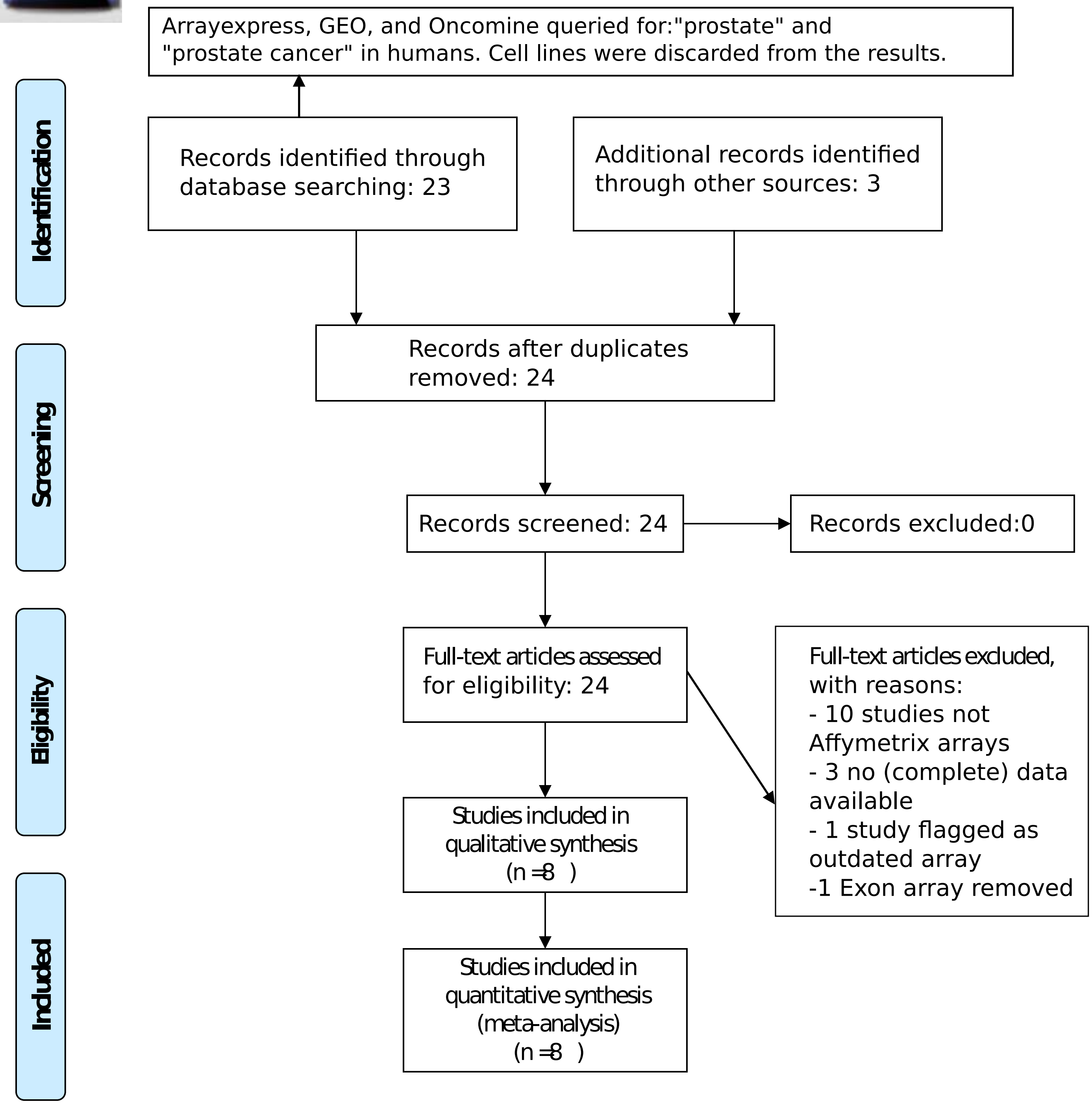

Supplementary Figure S1. Prisma Flow Diagram.

Modified *From:* Moher D, Liberati A, Tetzlaff J, Altman DG, The PRISMA Group (2009). *P*referred *R*eporting *I*tems for *S*ystematic Reviews and *M*eta-*A*nalyses: The PRISMA Statement. PLoS Med 6(6): e1000097. doi:10.1371/journal.pmed1000097

For more information, visit www.prisma-statement.org.
