# Supplementary material for: Characterization of Transcriptional Changes in ERG Rearrangement-Positive Prostate Cancer Identifies the Regulation of Metabolic Sensors Such as Neuropeptide Y
Source: PLoS One. 2013 Feb 4;8(2):e55207. doi: 10.1371/journal.pone.0055207 (PMC3563644; doi:10.1371/journal.pone.0055207)
Supplement: Figure S2 — Definition of ERG+ and ERG− prostate cancer tissues based on their ERG expression levels. (PDF) [file pone.0055207.s002.pdf]

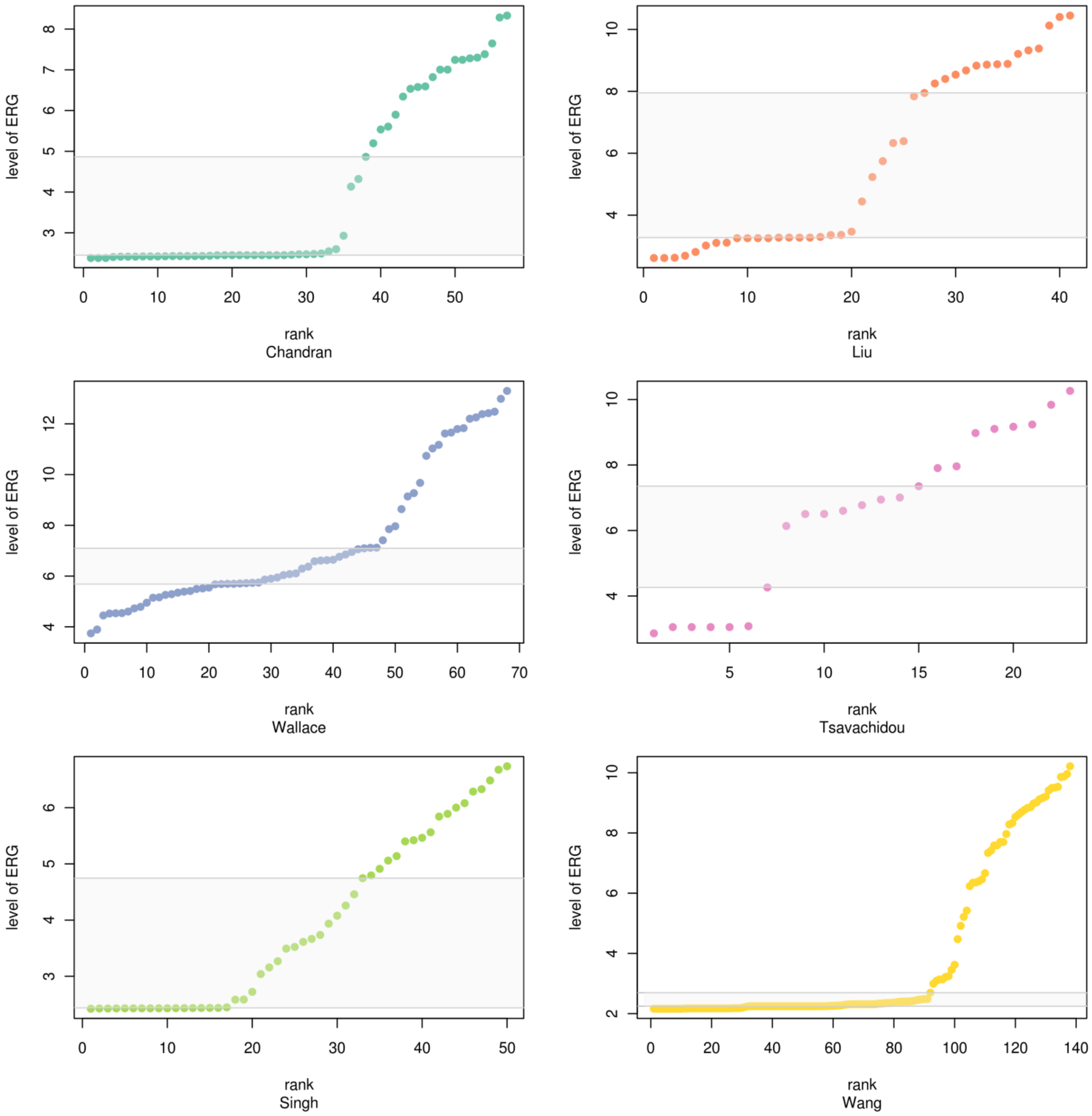

Supplementary Figure S2. Definition of ERG+ and ERG- prostate cancer tissues based on their ERG expression levels. ERG expression levels were assessed for every prostate cancer tissue included in the meta-analysis. All cancer tissues of one study were divided into 3 groups based on their ERG expression level: ERG+, ERG intermediate and ERG- tissues. ERG intermediate tissues (gray boxes) were excluded from further analyses. ERG+ tissues were compared to ERG- tissues. The studies performed by Bermudo et al. and Varambally et al. were excluded because of their small sample numbers (n=21 and n=7, respectively).
