# Supplementary material for: Characterization of Transcriptional Changes in ERG Rearrangement-Positive Prostate Cancer Identifies the Regulation of Metabolic Sensors Such as Neuropeptide Y
Source: PLoS One. 2013 Feb 4;8(2):e55207. doi: 10.1371/journal.pone.0055207 (PMC3563644; doi:10.1371/journal.pone.0055207)
Supplement: Figure S3 — ERG rearrangement results in ERG overexpression. (PDF) [file pone.0055207.s003.pdf]

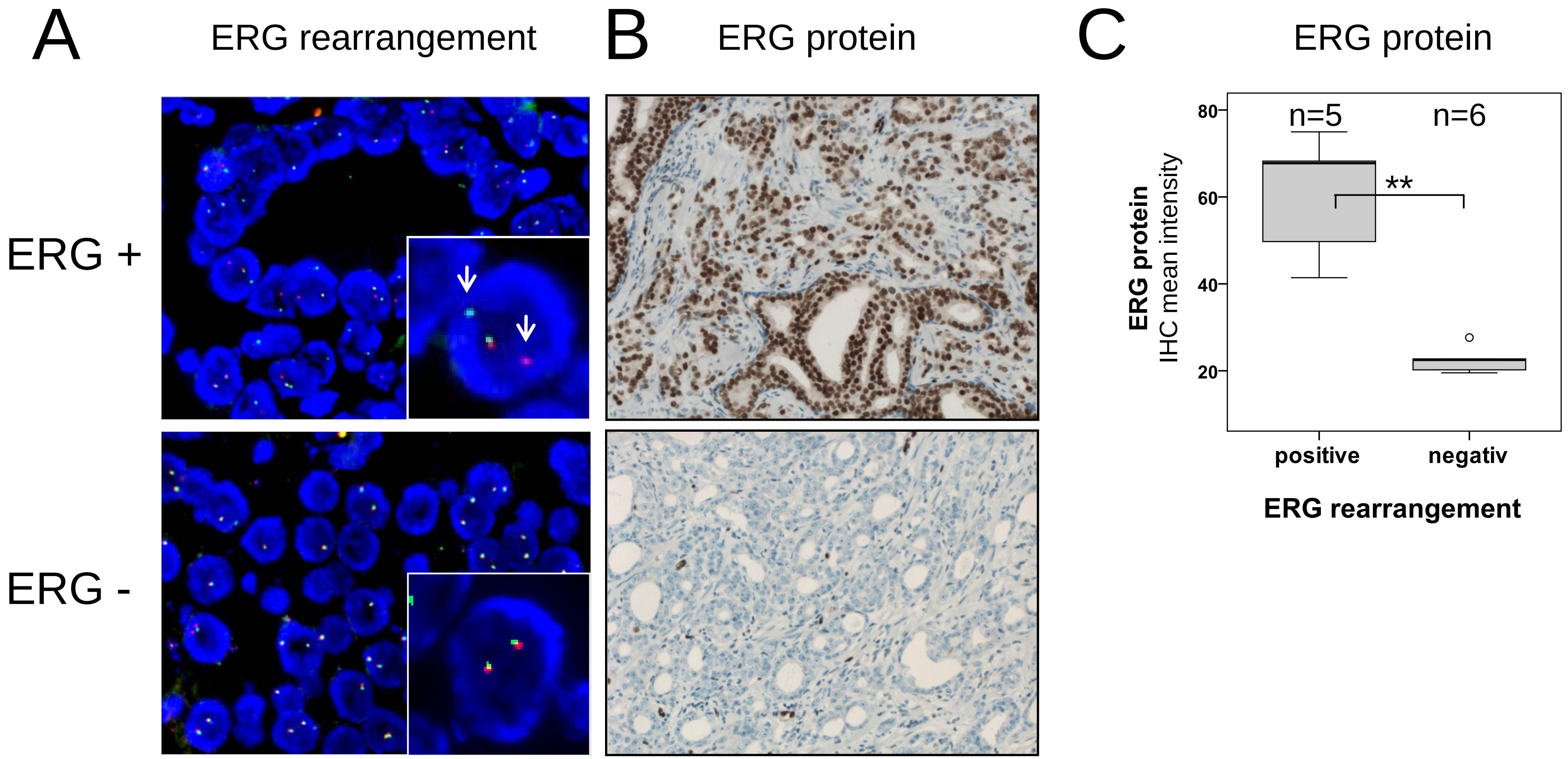

Supplementary Figure S3. ERG rearrangement results in ERG overexpression.

A) ERG fluorescence in situ hybridization using a break-apart assay. In ERG rearrangement-positive prostate cancer cells, the signal of the two fluorescent labeled probes breaks apart. In cells with ERG rearrangement (ERG+) the separation of red and green signals from one fusion indicates that the ERG gene has split apart. The remaining single fusion signal represents the normal (non-rearranged ERG) gene on the normal chromosome. **B)** ERG protein visualization using immunohistochemistry. ERG protein is over-expressed in ERG rearrangement-positive prostate cancer tissues. **C)** ERG protein quantification using the HistoQuest immunohistochemistry quantification program. Statistics, Mann Whitney U-test; *P<0.05; **P<0.01 ;***P<0.001.
