# Supplementary material for: Characterization of Transcriptional Changes in ERG Rearrangement-Positive Prostate Cancer Identifies the Regulation of Metabolic Sensors Such as Neuropeptide Y
Source: PLoS One. 2013 Feb 4;8(2):e55207. doi: 10.1371/journal.pone.0055207 (PMC3563644; doi:10.1371/journal.pone.0055207)
Supplement: Figure S4 — ERG immunohistochemistry. (PDF) [file pone.0055207.s004.pdf]

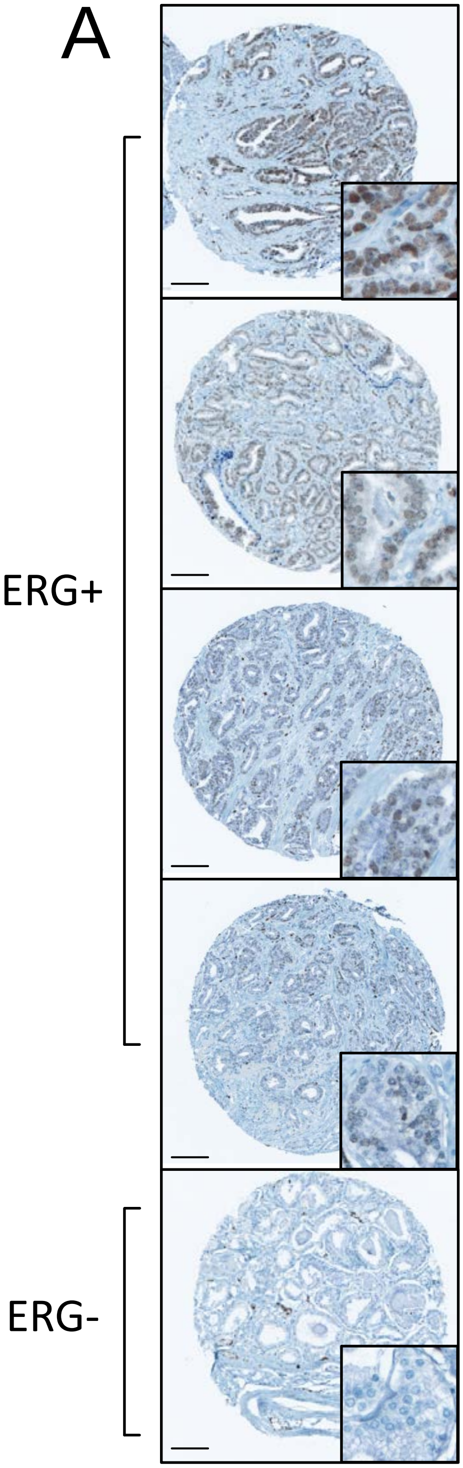

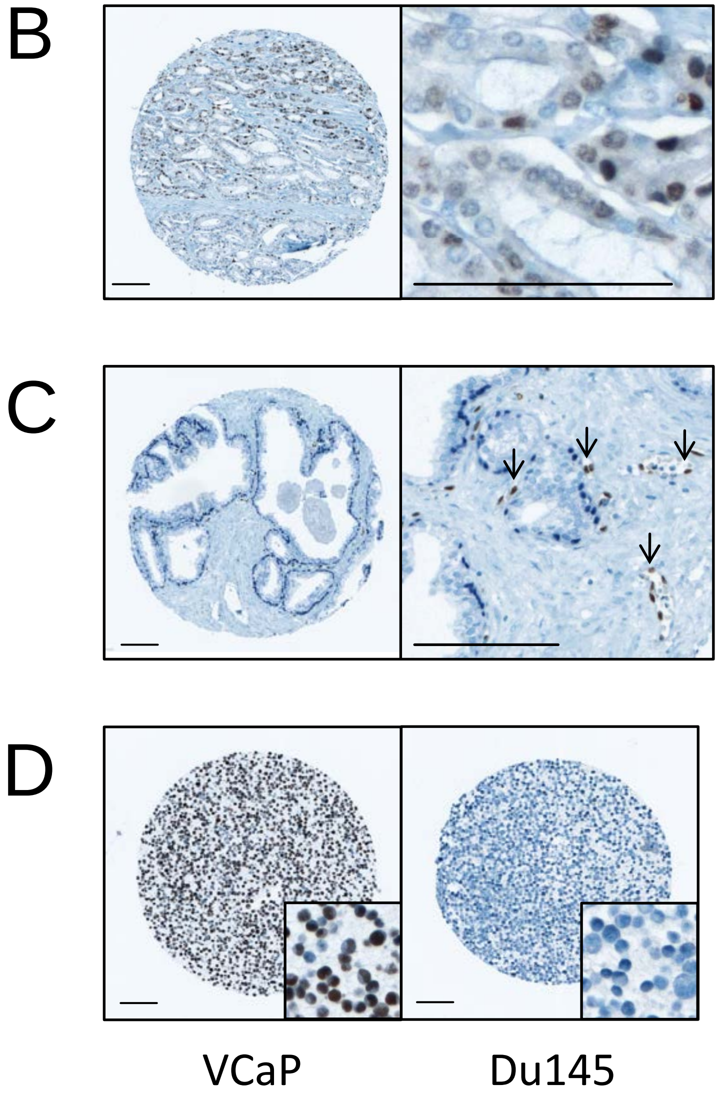

E

ERG IHC	staining intensity	n	%
positive	strong-intermediate	31	33.3
	weak	13	14.0
negative		38	40.9
heterogenous	various	11	11.8

Supplementary Figure S4. ERG immunohistochemistry. A Prostate cancer tissues showing different intensities of ERG staining. **B** Both ERG-positive and ERG-negative cancer cells were present in some cancer tissues. These were defined as heterogeneous ERG tissues. **C** Benign prostate tissues are ERG negative except for endothelial cells and lymphocytes (examples marked with arrows). **D** Controls were performed using ERG + (VCaP) and ERG - (Du145) cell lines. **E** Summary of 93 cancer cases used for this study.
Immunohistochemistry for ERG (brown) and p63 (marker for basal cells surrounding benign glands, blue). Bar, 100 μm.
