# Supplementary material for: Characterization of Transcriptional Changes in ERG Rearrangement-Positive Prostate Cancer Identifies the Regulation of Metabolic Sensors Such as Neuropeptide Y
Source: PLoS One. 2013 Feb 4;8(2):e55207. doi: 10.1371/journal.pone.0055207 (PMC3563644; doi:10.1371/journal.pone.0055207)
Supplement: Figure S5 — Expression of NPY and NPY-responsive receptors. (PDF) [file pone.0055207.s005.pdf]

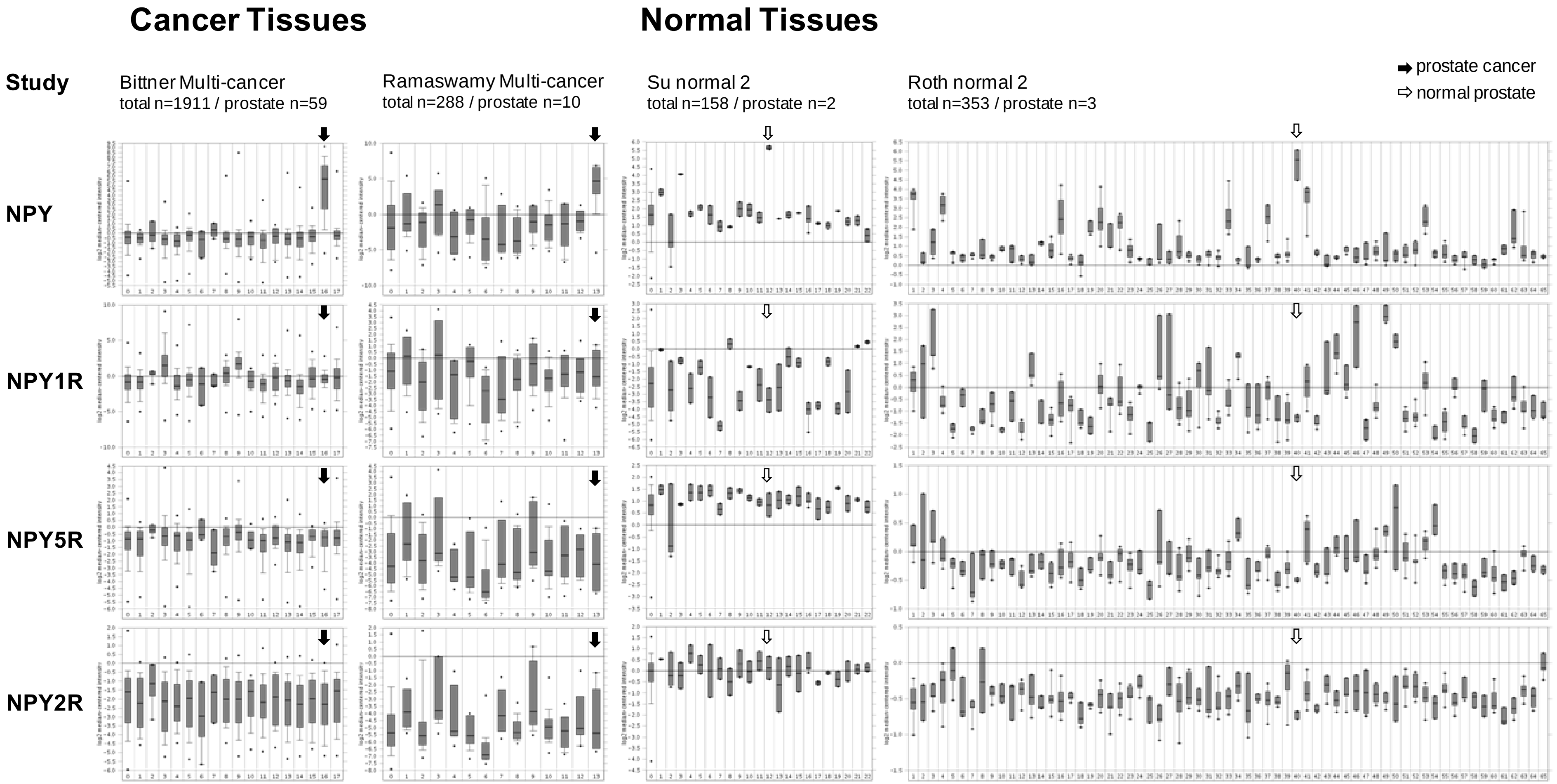

Legend

Bittner Multi-cancer	Ramaswamy Multi-cancer	Su normal 2	Roth normal		
0. No value (223)	0. No value (98)	0. No value (108)	1. Accumbens Nucleus (9)	24. Liver (4)	47. Skeletal Muscle Tissue (5)
1. Bladder Cancer (32)	1. Bladder Cancer (11)	1. Adrenal Gland (2)	2. Adipose Tissue (3)	25. Lung (3)	48. Spinal Cord (8)
2. Brain and CNS Cancer (4)	2. Brain and CNS Cancer (20)	2. Bone Marrow (6)	3. Adrenal Cortex (4)	26. Lymph Node (4)	49. Spleen (4)
3. Breast Cancer (328)	3. Breast Cancer (11)	3. Brain (2)	4. Amygdala (8)	27. Mammary Gland (3)	50. Subcutaneous Adipose Tissue (3)
4. Cervical Cancer (35)	4. Colorectal Cancer (11)	4. Heart (2)	5. Bone Marrow (5)	28. Medulla Oblongata (9)	51. Substantia Nigra (8)
5. Colorectal Cancer (330)	5. Kidney Cancer (11)	5. Kidney (2)	6. Bronchus (3)	29. Mesencephalon (9)	52. Subthalamic Nucleus (8)
6. Esophageal Cancer (7)	6. Leukemia (30)	6. Liver (2)	7. Buccal Mucosa (4)	30. Myometrium (5)	53. Temporal Lobe (8)
7. Gastric Cancer (7)	7. Lung Cancer (11)	7. Lung (2)	8. Gastric Cardia (3)	31. Nipple (4)	54. Testis (3)
8. Head and Neck Cancer (41)	8. Lymphoma (22)	8. Lymph Node (2)	9. Cardiac Atrium (4)	32. Nodose Ganglion (8)	55. Thalamus (8)
9. Kidney Cancer (254)	9. Melanoma (10)	9. Ovary (2)	10. Cardiac Ventricle (3)	33. Occipital Lobe (8)	56. Thyroid Gland (4)
10. Liver Cancer (11)	10. Other Cancer (21)	10. Pancreas (2)	11. Cecum (3)	34. Omental Adipose Tissue (4)	57. Tongue (4)
11. Lung Cancer (107)	11. Ovarian Cancer (11)	11. Pituitary Gland (2)	12. Cerebellum (9)	35. Ovary (4)	58. Tonsil (3)
12. Lymphoma (19)	12. Pancreatic Cancer (11)	**12. Prostate Gland (2)**	13. Cervix Uteri (4)	36. Papilla of the Tongue (4)	59. Trachea (3)
13. Other Cancer (220)	**13. Prostate Cancer (10)**	13. Salivary Gland (2)	14. Coronary Artery (3)	37. Parietal Lobe (9)	60. Trigeminal Ganglion (8)
14. Ovarian Cancer (166)		14. Skin (2)	15. Corpus Callosum (9)	38. Pharyngeal Mucosa (4)	61. Urethra (3)
15. Pancreatic Cancer (19)		15. Spinal Cord (2)	16. Cerebral Cortex (9)	39. Pituitary Gland (8)	62. Vagina (4)
**16. Prostate Cancer (59)**		16. Testis (4)	17. Dorsal Root Ganglion (8)	**40. Prostate Gland (3)**	63. Ventral Tegmentum (8)
17. Sarcoma (49)		17. Thymus Gland (2)	18. Endometrium (4)	41. Putamen (9)	64. Vestibular Nucleus (7)
		18. Thyroid Gland (2)	19. Esophagus (4)	42. Pylorus (4)	
		19. Tongue (2)	20. Frontal Lobe (9)	43. Renal Cortex (4)	
		20. Tonsil (2)	21. Fundus of the Stomach (4)	44. Renal Medulla (4)	
		21. Trachea (2)	22. Hippocampus (9)	45. Salivary Gland (4)	
		22. Uterus (2)	23. Hypothalamus (8)	46. Saphenous Vein (3)	

Supplementary Figure S5. Figure Legend on next page

Supplementary Figure S5. Expression of NPY and NPY-responsive receptors. NPY, NPY1R, NPY5R and NPY2R (NPY affinity ranked) expression in the prostate and other human organs was compared using the Oncomine database (http://www.oncomine.org). Four studies investigating normal and cancerous tissues are shown. Prostate tissues are highlighted with an arrow.
