# Supplementary material for: Characterization of Transcriptional Changes in ERG Rearrangement-Positive Prostate Cancer Identifies the Regulation of Metabolic Sensors Such as Neuropeptide Y
Source: PLoS One. 2013 Feb 4;8(2):e55207. doi: 10.1371/journal.pone.0055207 (PMC3563644; doi:10.1371/journal.pone.0055207)
Supplement: Methods S1 — Meta-analysis: preparation of data, statistical calculations. (DOC) [file pone.0055207.s008.doc]

**Supplementary material**

**Supplementary methods**

**Meta-analysis: preparation of data**

Quality assessment

We performed comprehensive quality controls of raw and preprocessed microarray data using Bioconductor's arrayQualityMetrics package , and excluded samples of suspicious quality from further analysis. The arrayQualityMetrics package provides extensive quality reports, including several diagnostic plots and measures to identify potential outlier arrays. It assesses individual array quality and between-array distances, as well as batch effects and spatial effects. Furthermore, it provides various specific quality assessment plots for microarrays of the Affymetrix platform.

Preprocessing

After removing outlier samples the raw data were normalized, adjusted for background noise, technical variability and non-specific binding, and summarized using the GCRMA (Guanine Cytosine Robust Multi-Array) algorithm . GCRMA is based on the RMA procedure, but additionally employs sequence-based probe affinity information, non-specific signal intensities of mismatch probes, and information across all given arrays to account for non-specific hybridization when adjusting for background noise. For normalization GCRMA utilizes quantile-based normalization, and for summarization the median polish algorithm, thus computing stabilized summary expression values for each gene from all available probes in their respective probe sets. Table 1 of supplementary methods summarizes the filtering of arrays. It lists the number of arrays that were flagged as being of low quality at different stages of the data preprocessing according to arrayQualityMetrics reports. The number of arrays that were used in the final analysis is given in Table 1 of the main text.

**Table 1 of supplementary methods.** The filtering summary displays the number of arrays that have been flag as being of low quality during the analysis. **Table 1 of supplementary methods.** The filtering summary displays the number of arrays and array names that have been flag as being of low quality during the analysis.

| **Study** | **Bad arrays (raw data)** | **Bad arrays (after processing)** |
| --- | --- | --- |
| Bermudo | 1  (1673907894) | 1  (1673907593) |
| Chandran | 9  (GSM152804, GSM152813, GSM152945, GSM152948, GSM152987, GSM152990, GSM187525, GSM187524, GSM152991) | 2  (GSM152810, GSM187526) |
| Liu | 3  (9763059872, 5642567629, 4418592762) | - |
| Singh | 4  (N55__normal, N60__normal, T01__tumor, T10__tumor) | - |
| Tsavachidou | 8  (1559552868, 1559552958, 1559553078, 1559553714, 1559553856, 1559553946, 1559554306, 1559554606) | 5  (1559552592, 1559552778, 1559552808, 1559553406, 1559554516) |
| Varambally | - | - |
| Wallace | 5  (GSM160420, GSM160422, GSM160428, GSM160430, GSM160431) | 2  (GSM160349, GSM160424) |
| Wang | 5  (GSM203310, GSM203375, GSM203376, GSM203377, GSM203378) | 2  (GSM203314, GSM203355) |

Mapping

To perform a cross-study comparison of gene expression levels, platform-specific gene probe-set identifiers were mapped to a common namespace, as previously described . Here the platform-specific identifiers were mapped to Entrez gene identifiers, using the current probeset/Entrez mappings from BioMart using the biomaRt package. The number of probesets and mapped EntrezGene identifiers are listed in table 2 of supplementary methods.

**Table 2 of supplementary methods.** Mapping between the original probeset identifiers and Entrez Gene. In case of a multiple match we used the probeset with the highest variance.

| **Study** | **Unique Probesets** | **Unique Entrez** | **Mapping Probe -> Entrez** | | **Mapping Entrez -> Probe** | |
| --- | --- | --- | --- | --- | --- | --- |
|  |  |  | **Unique** | **Multiple** | **Unique** | **Multiple** |
| Bermudo | 8306 | 8525 | 7978 | 328 | 8234 | 291 |
| Chandran | 10821 | 8983 | 10386 | 435 | 7058 | 1925 |
| Liu | 18229 | 12720 | 17471 | 758 | 8534 | 4366 |
| Singh | 10821 | 8983 | 17471 | 758 | 8534 | 4366 |
| Varambally | 30757 | 17959 | 29688 | 1069 | 9282 | 8677 |
| Wallace | 18229 | 12720 | 17471 | 758 | 8534 | 4366 |
| Wang | 18229 | 12720 | 17471 | 758 | 8534 | 436 |

**Meta-analysis: statistical calculations**

To derive a combined p-value across the different studies, we used Fisher's inverse chi-squared method . let i=1.I be a gene, then K(i) is the number of studies j= 1. J, where gene i was measured. For each gene we derived a p-value p(i,j) using a t-test. Following Fisher's method, a test statistic was calculated according the following equation:


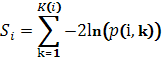
.

The corresponding p-value was derived from a chi-squared distribution with 2K(i) degrees of freedom . Using this method we calculated a p-value for each gene. To adjust for multiple testing we applied the Benjamini-Hochberg method . In addition to the p-value we derived a non-weighted and a weighted average log2 fold-change. For the weighted approach f(i,j) corresponds to the log2 fold change for gene i in study j. Based on sample size n(j) we calculated a weight for the contribution of each study as follows:


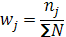
.

Note that relative weights depend on the number of studies in which this gene had been measured. Using these weights we calculated a weighted average. The weighted average is used in the paper. Both weighted and non-weighted averages are given in the online database at: http://prostatedb.eigenlab.net/. In addition to the less conservative Chi-squared method, which we applied for deriving a summary p-value , the database also lists a more conservative summary p-value derived by a permutational approach .

**References for supplementary methods**

1. Kauffmann A, Gentleman R, Huber W (2009) arrayQualityMetrics--a bioconductor package for quality assessment of microarray data. Bioinformatics 25: 415-416.

2. Wu Z, Irizarry RA (2005) Stochastic models inspired by hybridization theory for short oligonucleotide arrays. J Comput Biol 12: 882-893.

3. Wu Z, Irizarry R, Gentleman R, Martinez-Murillo F, Spencer F (2004) GCRMA A Model-Based Background Adjustment for Oligonucleotide Expression Arrays. Journal of the American Statistical Association 99: 909.

4. Rhodes DR, Barrette TR, Rubin MA, Ghosh D, Chinnaiyan AM (2002) Meta-analysis of microarrays: interstudy validation of gene expression profiles reveals pathway dysregulation in prostate cancer. Cancer Res 62: 4427-4433.

5. Romualdi C, De Pitta C, Tombolan L, Bortoluzzi S, Sartori F, et al. (2006) Defining the gene expression signature of rhabdomyosarcoma by meta-analysis. BMC Genomics 7: 287.

6. Fisher RA (1925) Statistical Methods for Research Workers. Edinburgh: Oliver & Boyd

7. Moreau Y, Aerts S, De Moor B, De Strooper B, Dabrowski M (2003) Comparison and meta-analysis of microarray data: from the bench to the computer desk. Trends Genet 19: 570-577.

8. Benjamini Y HY (1995) Controlling the false discovery rate: A practical and powerful approach to multiple testing. J of Royal Statistical Society B 57: 289-300.
